# Proposal for Practical Approach in Prenatal Diagnosis of Beckwith–Wiedemann Syndrome and Review of the Literature

**DOI:** 10.3390/diagnostics12071709

**Published:** 2022-07-13

**Authors:** Gwo-Chin Ma, Tze-Ho Chen, Wan-Ju Wu, Dong-Jay Lee, Wen-Hsiang Lin, Ming Chen

**Affiliations:** 1 Department of Genomic Medicine and Center for Medical Genetics, Changhua Christian Hospital, Changhua 50046, Taiwan; 128729@cch.org.tw (G.-C.M.); crystalwu835@gmail.com (W.-J.W.); 2 Research Department, Changhua Christian Hospital, Changhua 50006, Taiwan; 118862@cch.org.tw; 3 Department of Medical Laboratory Science and Biotechnology, Central Taiwan University of Science and Technology, Taichung 40601, Taiwan; 4 Department of Obstetrics and Gynecology, Changhua Christian Hospital, Changhua 50006, Taiwan; 46305@cch.org.tw; 5 Welgene Biotechnology Company, Nangang Business Park, Taipei 11560, Taiwan; 397620cch@gmail.com; 6 Department of Medical Genetics, National Taiwan University Hospital, Taipei 10041, Taiwan; 7 Department of Obstetrics and Gynecology, College of Medicine, National Taiwan University, Taipei 10041, Taiwan; 8 Department of Medical Science, National Tsing Hua University, Hsinchu 30013, Taiwan; 9 Department of Biomedical Science, Da-Yeh University, Changhua 51591, Taiwan; 10 Department of Post-Baccalaureate Medicine, College of Medicine, National Chung Hsing University, Taichung 40227, Taiwan

**Keywords:** BWS, ultrasonography, imprinting, uniparental disomy, MS-MLPA, ICR1, ICR2, omphalocele

## Abstract

Background: Beckwith–Wiedemann syndrome (BWS) is a phenotypically and genetically heterogeneous disorder associated with epigenetic/genetic aberrations on chromosome 11p15.4p15.5. There is no consensus criterion for prenatal diagnosis of BWS. Methods: Three BWS patients with their clinical histories, prenatal ultrasonographic features, and results of molecular diagnosis were presented. Likewise, by incorporating the findings of our cases and literature review, the phenotypic spectrum and genotype–phenotype correlations of fetal BWS were summarized, and a practical approach in prenatal diagnosis of BWS was proposed. Results: A total of 166 BWS cases with prenatal features were included for analysis. Common fetal features include abdominal wall defects (42.8%), polyhydramnios (33.1%), and macrosomia (32.5%). Molecular pathologies include methylation changes in imprinting control region 1 and 2 (ICR1 and ICR2), paternal uniparental disomy of chromosome 11p15.5, copy number change involving 11p15, etc. Some genotype–phenotype correlations were observed. However, the broad phenotypic spectrum but limited features manifested by affected fetuses rendering ultrasonographic diagnosis not easy. Conclusions: Molecular tests are used for prenatal diagnosis of BWS suspected by ultrasonography. Methylation-specific multiplex ligation-dependent probe amplification (MS-MLPA) is recommended as the first-line molecular tool because it simultaneously detects ICR1/ICR2 methylation statuses and copy numbers that solve the majority of clinical cases in the prenatal scenario.

## 1. Introduction

Beckwith–Wiedemann syndrome (BWS) is a growth malformation disorder that affects multiple organ systems and manifests variable expressivity. Features related to BWS include macrosomia, macroglossia, abdominal wall defects (omphalocele, umbilical hernia, and diastasis recti), visceromegaly (kidneys, liver, spleen, pancreas, and adrenal glands), facial abnormalities (ear creases and pits, facial nevus flammeus, metopic ridge), hemihypertrophy, and hypoglycemia [1,2]. Patients with BWS also face an increased risk of childhood malignancies (Wilms’ tumor, neuroblastoma, adrenal carcinoma, hepatoblastoma, rhabdomyosarcoma) [3]. The incidence of BWS is estimated to be 1 out of 10,000 live births [4], with increased risk associated with assisted reproductive technology (ART) of about 1 in 1100 [5].

**Figure 1:**
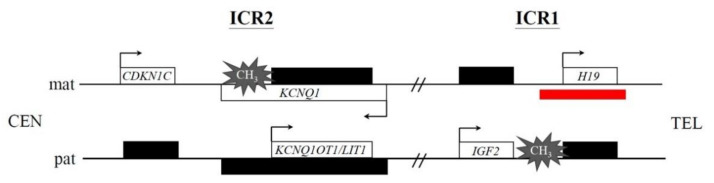
Molecular etiology of Beckwith–Wiedemann syndrome (BWS). Schematic representation of the two neighboring imprinted domains at human chromosome 11p15.4p15.5, namely imprinting control region 1 (ICR1) and ICR2, related to BWS. Active gene indicated by white symbol, inactive gene by black symbol, differentially methylated region by gray symbol, and direction of transcription by arrow. The maternal and paternal alleles by “mat” and “pat”, respectively. CEN, centromere; TEL, telomere. The red bar indicates the 11p15.5 deletion detected in our Patient 3 (case III-9 in Figure 2) that covers the ICR1 of the maternal allele.

The molecular mechanism underlying BWS is also heterogeneous and associated with both of epigenetic and genetic alternations on chromosome 11p15.4p15.5. The region harbors a cluster of imprinted genes and is functionally divided into two domains: the telomeric and centromeric domains. The telomeric domain includes the long intergenic noncoding RNA H19 (*H19*) and insulin-like growth factor 2 (*IGF2*) genes [6,7,8] that are regulated by the imprinting control region 1 (ICR1), also known as imprinting center 1 (IC1) or differentially methylated region 1 (DMR1) (Figure 1). The paternal ICR1 is imprinted by methylation that prevents *H19* activation but permits *IGF2* expression. In contrast, the maternal ICR1 is unmethylated and allows *H19* activation but silences *IGF2* expression (Figure 1). The centromeric domain includes the cyclin-dependent kinase inhibitor 1C (*CDKN1C*), potassium voltage-gated channel subfamily Q member 1 (*KCNQ1*), and *KCNQ1*-overlapping transcript 1 (*KCNQ1OT1*) (also known as long QT intronic transcript 1, *LIT1*) genes [9,10,11] that are coordinated by the ICR2/IC2/DMR2 (Figure 1). The maternal ICR2 is methylated and prevents *KCNQ1OT1* activation but permits *CDKN1C* and *KCNQ1* expression. On the contrary, the paternal ICR2 is unmethylated and allows *KCNQ1OT1* activation but silences *CDKN1C* and *KCNQ1* expression (Figure 1). Methylation alternation, point mutation, deletion, duplication, and chromosomal rearrangement involving the centromeric domain are causative of 85% of BWS patients, while molecular anomalies in the telomeric domain are causative of 5–7% of BWS cases [2,12,13,14]. Overall, ICR2 hypomethylation is the most common cause found in 50–60% of patients, followed by paternal uniparental disomy chromosome 11 (patUPD11) and ICR1 hypermethylation, detected in 20–25% and 5–10% of cases, respectively [15]. *CDKN1C* mutations are found in about 5% of sporadic BWS and 40% of familial cases [15].

Patients with BWS are commonly diagnosed via clinical and pathologic investigation. Different systems with various combinations of clinical features have been proposed to define BWS [2,16,17]. The consortium of European Network of Human Congenital Imprinting Disorders (EUCID.net) supports a clinical scoring system that classifies the phenotypic abnormalities into cardinal features (e.g., macroglossia, omphalocele) and suggestive features (e.g., birthweight > 2 standard deviation scores above the mean, facial nevus simplex) [2]. Each of the cardinal and suggestive features is scored as 2 and 1 points, respectively. Patients with scoring value ≥ 4 are clinically diagnosed as BWS, while patients with scoring value < 2 are not recognized as BWS cases [2].

In contrast to postnatal cases that can be diagnosed by phenotypic scoring, prenatal diagnosis of BWS is relatively challenging because some cardinal features cannot be detected by ultrasonography (e.g., hemihyperplasia), and some features appearing after GA of 30 weeks (e.g., macrosomia and macroglossia) could be missed in second trimester fetal structural screening [18]. Currently, treatments are available for some of the symptoms of BWS (e.g., abdominal wall repair, surgical tongue reduction), but neonatal death may occur due to complications of macroglossia, cardiomyopathy, prematurity, or hypoglycemia [19]. Prenatal recognition of BWS is helpful for monitoring and timely treatment of complications after birth.

Molecular examination provides an alternative method for prenatal diagnosis of this complex disorder. Here, we report the clinical history and prenatal ultrasonographic findings of three BWS cases. The gross appearance and autopsy of aborted fetuses showed compatible findings to the prenatal ultrasonography and met the diagnostic criteria of BWS. Molecular diagnosis confirmed that all the three cases are caused by genetic/epigenetic defects in chromosomal 11p15 region. By incorporating the clinical findings of these cases and the literature review, we further summarize the phenotypic spectrum and genotype–phenotype correlations of fetal BWS and propose a practical approach to facilitate the prenatal diagnosis of BWS.

## 2. Materials and Methods

### 2.1. Patients

**Figure 2:**
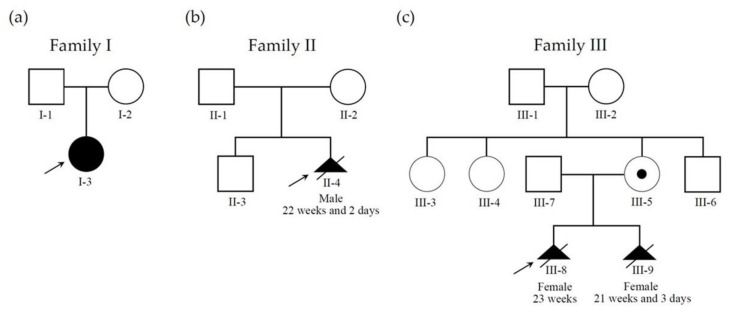
Pedigree information of three families with BWS cases. (**a**) Family I has a sporadic BWS child (case I-3; Patient 1) who was diagnosed as hypomethylation at maternal ICR2 on chromosome 11p15.5 region. (**b**) Family II has a sporadic BWS fetus (case II-4; Patient 2) who was diagnosed as hypomethylation at maternal ICR2. The parents accepted termination of pregnancy (TOP) at gestation age (GA) = 22 weeks and two days. (**c**) Family III has two consecutive BWS fetuses (case III-8 and III-9; III-9 is Patient 3). Both cases are diagnosed to have a maternal derived chromosome 11p15.5 deletion involving in ICR1 and *H19*, resulting in hypermethylation in ICR1. The pregnant woman (III-5) carries a deletion on chromosome 11p15.5 in a mosaic status without phenotypic abnormalities. The parents opted TOP for both fetuses (case III-8 and case III-9) at GA = 23 weeks and 21 weeks and three days, respectively. Male indicated by square, female by circle, carrier by a dot in the middle of the symbol, affected individual by filled symbol, TOP by triangle with a slash, and proband by arrow.

Three pregnant women (from three unrelated families) who visited our hospital from 2018 to 2019 were enrolled in this study (case I-2, II-2, and III-5 in Figure 2). These women underwent amniocentesis in the second trimester due to abnormal ultrasound findings of their singleton pregnancies. Genetic analyses were performed for the three fetuses, of which one was confirmed of BWS after birth (Patient 1; case I-3 in Figure 2), and the remaining two were prenatally diagnosed as BWS (Patient 2 and 3; case II-4 and III-9, respectively, in Figure 2).

### 2.2. DNA Extraction

DNAs from amniocytes and peripheral blood leukocytes were extracted using Puregene Extraction Kit (Qiagen, Hilden, Germany). DNA quality and purity were evaluated based on the values and ratio of the absorbances at 260 nm and 280 nm using ND-1000 spectrophotometer (Labtech International, East Sussex, UK).

### 2.3. Cytogenetic Analysis

Chromosomal compositions were examined by cytogenetic analysis. The amniocytes were cultured in amniocyte culture medium BIO-AMF-1 (Biological Industries, Cromwell, CT, USA) and grew in an incubator at 37 °C with 5% CO_2_ for 9 to 10 days. The cells were harvested when multiple clones with metaphase cells were observed under an inverted microscope. Conventional G banding was performed with Wright’s dye staining. Twenty chromosome karyotypes were counted, and seven karyotypes were analyzed.

### 2.4. Chromosome Microarray Analysis (CMA)

Copy number analyses were performed by CMA using an Agilent customer design oligonucleotide 8 × 60 K CytoScan^®^ gene chip (ID 040427). DNA labeling and hybridization were carried out according to manufacturer’s recommendation. Scanned images were analyzed by Feature Extraction 9.5.3 software (Agilent Technologies, Santa Clara, CA, USA), and the extracted data were processed using the Agilent Genomic Workbench 7.0 program (Agilent Technologies, CA, USA). The CMA findings were described based on the reference genome version of GRCh37, following the latest guideline of An International System for Human Cytogenomic Nomenclature (ISCN2020).

### 2.5. Methylation-Specific Multiplex Ligation-Dependent Probe Amplification (MS-MLPA)

DNA methylation of ICR1 and ICR2 and the imprinted gene dosage of *H19* and *KCNQ1OT1/LIT1* of chromosome 11p15 were analyzed by MS-MLPA according to the instruction of the manufacture (ME030-C3 BWS/RSS, MRC-Holland, Amsterdam, The Netherlands). The MS-MLPA products were run on GenomeLab™ GeXP Genetic Analysis System (Beckman Coulter Inc., Brea, CA, USA), and the data were collected by FRAGMENTS application program (Beckman Coulter Inc.). The collected data were analyzed using an in-house-designed Excel-based program that is able to perform all normalization steps.

### 2.6. Literature Review

The *PubMed* and *Web of Science* databases were searched through 17 March 2022, along with grey literature and reference list searches. The search strategies and keywords used are as follows: “Beckwith–Wiedemann syndrome”, “BWS”, “prenatal”, “ultrasound”, “genetic diagnosis”, “fetal feature”, “macrosomia”, “macroglossia”, “visceromegaly”, “hemihypertrophy”, “abdominal wall defects”, “ICR2 hypomethylation”, “ICR1 hypermethylation”, “patUPD11p15”, and “uniparental disomy”. As we focused on the study of the prenatal cases, studies exclusively concerning postnatal findings, clinical management, and evolution of postnatal diagnostics were excluded.

## 3. Results

### 3.1. Patients

Three BWS patients from three unrelated families were included in this study, of which one was confirmed of BWS after birth (Patient 1 is case I-3 in Figure 2), and the remaining two were prenatally diagnosed as BWS (Patient 2 and 3 are case II-4 and III-9, respectively, in Figure 2).

#### 3.1.1. Patient 1

**Figure 3:**
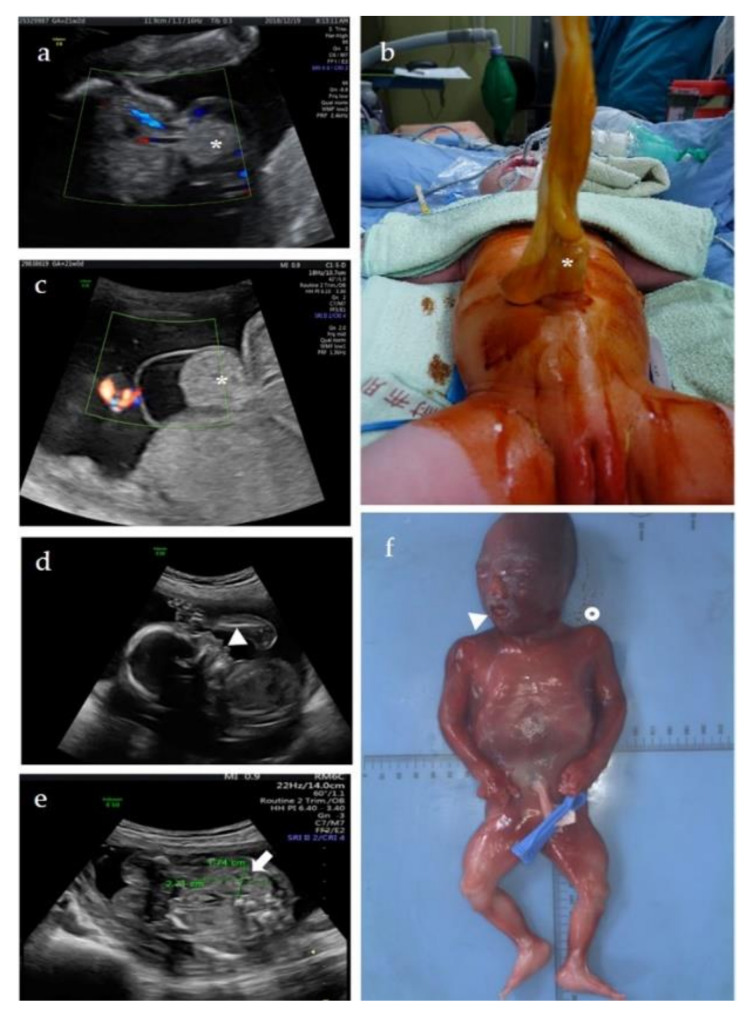
Clinical features of three patients with BWS. Patient 1 (**a**,**b**): Prenatal ultrasonography identified (**a**) isolated umbilical hernia (star) at GA = 21 weeks and two days. The position of cord insertion was normal and protruding small intestine was noted. (**b**) The umbilical hernia with 3 cm abdominal wall defect and small bowel protruding (star) was identified after birth (GA = 38 weeks). Macrosomia with body weight of 3850 g (96th percentile) was also noted. Patient 2 (**c**): Prenatal ultrasonography identified (**c**) isolated omphalocele (star) at GA = 21 weeks. The cord inserted on the apex of herniated sac. Patient 3 (**d**–**f**): Prenatal ultrasonography identified (**d**) protruding tongue (triangle) and (**e**) unilateral nephromegaly (fetal kidney length: 2.71 cm and transverse diameter: 1.74 cm; both values > 95th percentile) (arrow) at GA = 20 weeks and two days. (**f**) Macroglossia (triangle), board nose, and hemihyperplasia (circle) were further identified after TOP at GA = 21 weeks and 3 days.

A 31-year-old Taiwanese woman who naturally conceived, gravida 1, para 0, received prenatal care at our center since the first trimester. Isolated umbilical hernia (Figure 3a) was found at GA = 21 weeks and two days, but otherwise, no other structural abnormality was noted. The estimated fetal weight was 460 g (79th percentile). BWS was not speculated due to our inexperience of fetal BWS diagnosis then. Cytogenetic analyses and chromosome microarray analysis (CMA) were offered to detect if any chromosomal/genomic variants associated with the fetal anomaly. Results of both tests showed a normal female karyotype without pathogenic copy number variations (CNVs), 46,XX.arr(X,1−22)×2. The parents decided to continue the pregnancy, and the pregnancy was uneventful until birth. A female baby was delivered at GA = 38 weeks by cesarean section. The birth body weight was 3850 g (96th percentile), and Apgar score after birth were 8 and 9 in the first minute and fifth minutes, respectively. Macrosomia, in addition to umbilical hernia with 3 cm abdominal wall defect and small bowel protruding (Figure 3b), was noted, leading to suggest of BWS. However, neither neonatal hypoglycemia nor asymmetric extremities was found. Neonatal abdominal ultrasonography revealed normal size of kidney and liver without intraabdominal tumor. Surgery of umbilical hernia repair with small bowel reduction was arranged at the first day after birth, and the baby was discharged without complication a few days later. Methylation-specific multiplex ligation-dependent probe amplification (MS-MLPA) test of neonatal peripheral blood for BWS was performed, and the result showed ICR2 hypomethylation (8.3%) on chromosome 11p15, confirming the diagnosis of BWS. The methylation status in ICR1 was within the normal range (48.9%). No copy number change was detected in ICR1 and ICR2.

#### 3.1.2. Patient 2

A 32-year-old Taiwanese woman who natural conceived, gravida 3, para 1, visited our center because of abnormal prenatal ultrasonography at GA = 21 weeks. The fetal weight was estimated as 395 g (47th percentile). Detailed fetal ultrasonography revealed isolated omphalocele (Figure 3c). Patient 2 was suspected as a fetal BWS based on the diagnostic experience of Patient 1. Amniocentesis was carried out for CMA and MS-MLPA to detect the copy number and methylation status of the ICR2 and ICR1 regions. CMA revealed a normal male genomic composition (arr(X,Y)×1,(1−22)×2), but MS-MLPA revealed ICR2 hypomethylation (10.6%), confirming the diagnosis of BWS. The methylation status in ICR1 was normal, and no copy number change was detected in ICR1 and ICR2. MS-MLPA performed for the parents showed normal copy number and methylation status in ICR2 and ICR1. After nondirective counseling, pregnancy was terminated at GA = 22 weeks and two days, and a 520 g (63rd percentile) male fetus was aborted. Omphalocele was confirmed for the abortus, but an autopsy was denied by the couple.

#### 3.1.3. Patient 3

A 37-year-old Taiwanese woman, gravida 2, para 1, visited our center for genetic counseling at GA = 10 weeks. According to the medical record, the fetus of her first pregnancy was found to have a microdeletion on chromosome 11p15.5, including the ICR1 (Figure 1), by CMA with indication of anxiety. The fetus was subsequently diagnosed as BWS at GA = 22 weeks by MS-MLPA that showed full methylation (100%) in ICR1. The pregnancy was terminated at GA = 23 weeks.

During this pregnancy, chorionic villus sampling (CVS) was provided at GA = 12 weeks. The crown-rump length of the fetus was 63.2 mm (estimated GA = 12 weeks and 5 days). Chromosomal analysis revealed a normal female 46,XX karyotype. CMA identified a 32 kb deletion on chromosome 11p15.5, (arr[GRCh37] 11p15.5(1996741_2028877)×1), identical to that found in the first pregnancy (Figure 1). Parental follow-up showed the 11p15.5 deletion is of maternal origin. The mother carried the 11p15.5 deletion in mosaicism (arr[GRCh37] 11p15.5(1996741_2028877)×1~2) without phenotypical abnormality. MS-MLPA of the villi, maternal blood, and paternal blood revealed 1, 1.53, and 2 copies in the *H19* gene, respectively. The methylation status of the ICR1 in the villi, maternal blood, and paternal blood are hypermethylation (98.2%), hypomethylation (37.5%), and normal methylation (50%), respectively. The copy number and methylation status of villi and bloods of both parents in ICR2 were normal. The fetus was diagnosed with BWS. Detailed ultrasonography performed at GA = 20 weeks and two days showed protruding tongue (macroglossia) (Figure 3d) and nephromegaly, in which both fetal kidney length (2.71 cm) and transverse diameter (1.74 cm) were above 95th percentile (Figure 3e) [20]. The couple decided to terminate the pregnancy. TOP was performed at GA = 21 weeks and 3 days, and a 585 g (> 97th percentile) female fetus was aborted. Postmortem examination showed macroglossia, a broad nose, clear anterior ear creases, visceromegaly (including lung, kidney, and adrenal gland), and hemihyperplasia (Figure 3f).

### 3.2. Literature Review

A total of 166 BWS patients with prenatal features were reported, including the 3 cases present in this study, 50 fetal BWS [21,22,23,24,25,26,27,28,29,30,31,32,33,34,35,36,37,38,39,40,41,42,43,44,45,46,47], and two cohorts of patients that include 24 [48] and 89 BWS cases [49], respectively (Table S1). BWS was diagnosed prenatally in 56 fetuses, of which outcomes were available in 31 cases (55.4%; 31/56), consisting of 16 live births (51.6%; 16/31), 14 TOP (45.2%; 14/31), and 1 of in-utero demise (3.2%; 1/31) (Table S1). BWS was diagnosed postnatally in 110 cases; their prenatal findings were retrospected.

The fetal manifestations of BWS vary and include umbilical hernia, omphalocele, macroglossia, protruding tongue, macrosomia, polyhydramnios, short femurs, corpus callosum anomaly, intrauterine growth restriction, nephromegaly, hepatomegaly, cardiomegaly, liver and placental cystic lesion, diaphragmatic hernia, echogenic bowel, heat defects, intra-abdominal cyst, tumors, etc. (Table S1). Abdominal wall defects (e.g., umbilical hernia, omphalocele) are the most prevalent prenatal sign detected in 42.8% (71/166) of cases (Table 1), which is followed in order by polyhydramnios (33.1%; 55/166), macrosomia (32.5%; 54/166), macroglossia (18.1%; 30/166), organomegaly (e.g., nephromegaly, hepatomegaly, cardiomegaly) (17.5%; 29/166), placentomegaly (7.8%; 13/166), tumor (e.g., placental tumor, macroglossia, intracardiac rhabdomyoma) (3.6%; 6/166), and corpus callosum anomaly (1.2%; 2/166) (Table 1). In the 53 fetuses whose clinical features were available individually (i.e., case I-53 in Table S1), the types of abdominal wall defects can be further classified into isolated and nonisolated umbilical hernia/omphalocele that were identified in 26.4% (14/53) and 41.5% (22/53) of cases, respectively. Other isolated findings include mesenteric cystic lesion, intracardiac tumor, and renal cystic lesion; each was reported in one case (see cases 16, 19, and 27 in Table S1).

In 154 cases out of 166 with prenatal findings, molecular pathologies were reported, including ICR1 hypermethylation, ICR2 hypomethylation, patUPD11p15.5, chromosomal 11p15 abnormalities (including deletion, duplication, and rearrangement), and *CDKN1C* mutation (Table S1). Overall, ICR2 hypomethylation is the most common causation, accounting for 59.6% (99/166) of cases (Table 1). The patUPD11p15.5 and ICR1 hypermethylation follow in decreasing order, which were detected in 19.3% (32/166) and 8.4% (14/166) of cases, respectively (Table 1). Cases caused by unknown or other genetic defects (e.g., chromosome deletion/duplication/rearrangement and *CDKN1C* mutation) accounted for 12.7% (21/166).

For fetuses with ICR2 hypomethylation, abdominal wall defects, especially the omphalocele, are the most common feature detected in 57.6% (57/99) of cases. Other common features include polyhydramnios (33.3%; 33/99) and macrosomia (26.3%; 26/99) (Table 1). For fetuses with ICR1 hypermethylation, organomegaly, macrosomia, polyhydramnios, and macroglossia were prevalent, and each was found in 57.1% (8/14), 50.0% (7/14), 50.0% (7/14), and 42.9% (6/14) of cases, respectively (Table 1). For fetuses with patUPD11p15.5, macrosomia is the most common sign and was detected in 34.4% (11/32) of cases (Table 1).

## 4. Discussion

Though fetal manifestations, such as macrosomia, macroglossia, and abdominal wall defects, have been suggested for prenatal diagnosis of BWS [1], the display of these features in affected fetuses are rather variable, and none of the features detectable in utero are uniquely pathognomonic [2], making clinical diagnosis of fetal BWS sometimes difficult. Recently, molecular analysis has provided a remarkable advance in postnatal confirmation of BWS and may be also feasible for prenatal diagnosis of this complex disease [2,50]. Understanding the phenotypic spectrum of fetal BWS is crucial for the recognition of fetuses suspected with BWS in whom molecular diagnostic investigation is considered.

In our cases, Patient 1 was a sporadic case who was not diagnosed prenatally but after birth. Isolated umbilical hernia was found prenatally, but BWS was suspected due to the manifestations of macrosomia and umbilical hernia in the newborn baby. The syndrome was confirmed by MS-MLPA test that showed ICR2 hypomethylation. Patient 2 was also a sporadic case but was diagnosed prenatally. This case presented with isolated omphalocele and showed ICR2 hypomethylation. Patient 1 and 2 exemplified a diagnostic challenge of fetal BWS because the prenatal feature included abdominal wall defect only. In fact, abdominal wall defect is a relatively common feature of fetal BWS [18], but the absence of other associated findings rendered it difficult for differential diagnosis. Patient 3 was a rarely familial case diagnosed prenatally. CMA identified a maternal-origin 32 kb deletion on chromosome 11p15.5, including the ICR1. MS-MLPA confirmed the chromosome 11p15.5 deletion and identified ICR1 hypermethylation consistent with the molecular diagnosis of BWS. The case showed BWS features of macroglossia and nephromegaly during the pregnancy. Hemihyperplasia was then identified after abortion. Most cases of BWS are sporadic, accounting for approximately 85%, and only approximately 15% of cases have a family history, following a parent-to-child inheritance [19].

The pregnant woman of Patient 3 deserves a further discussion because she showed normal phenotype although she carried the 32 kb deletion on chromosome 11p15.5 in mosaic status. Mosaic genetic changes frequently lead to a range of clinical phenotypes depending on the population of cells affected [51]. No symptom in the pregnant woman may be attributed to the presence of a sufficient proportion of normal cells (without the 11p15.5 deletion) in tissues. Besides, levels of mosaicism can change over time. It has been shown that the BWS features become unapparent with age, and expressions of BWS in transmitting parents are variable and may be unrecognized [16]. Nevertheless, deletion in the paternal allele seems more rational because ICR1 hypomethylation was evidenced in the pregnant woman. It is a pity that we did not test further the parents of this pregnant woman due to unfeasibility. When the pregnant woman transmitted the deletion to her baby, the ICR1 methylation profile changed from hypomethylation to hypermethylation, which contributed to the syndrome of the fetus. It has been shown that maternal transmission of familial BWS is related to a more severe phenotype than paternal transmission [16,52]. Prenatal CMA is beneficial in families with BWS history associated with 11p15.5 deletion (e.g., Patient 3) [53,54]. Our experience further highlighted the need of applying more molecular tests when copy number variations (CNVs) detected involved specific regions such as 11p15.5. Noninvasive prenatal screening (NIPS) based on cell-free fetal DNA (cffDNA) testing is certainly not directly helpful in diagnosing BWS unless by detecting UPD11 and resulting in the subsequent related analyses [55]. The NIPS result did not truly reflect the fetal condition because the majority of cffDNA in maternal blood is of placental origin [56]. Future promising reproductive options, such as preimplantation genetic diagnosis (PGD), can also be offered to select those embryos not carrying the mutant allele derived from parents and then avoid recurrent of the disease [57]. A successful pregnancy by PGD to prevent passing on a maternal *CDK1C* mutation and thus BWS to offspring was recently reported [58].

Features that can be detected prenatally by ultrasound may be suggested for the syndrome. To understand the phenotypic spectrum and feature frequency of fetal BWS, we reviewed 166 patients, encompassing almost all the reported BWS with prenatal features so far. Abdominal wall defects are the most prevalent fetal sign detected in 42.8% of cases. These abnormalities are easily and can be early identified with ultrasonography. More than 90% fetuses with omphalocele, for example, were diagnosed in the first or second trimester of pregnancy [59]. BWS was considered to be more common with isolated omphalocele compared to nonisolated omphaloceles, which were frequently related to aneuploidy [59,60]. Our results showed that nonisolated omphalocele/umbilical hernia is also commonly seen in fetal BWS (nonisolated and isolated omphalocele/umbilical hernia were reported in 41.5% and 26.4% of cases, respectively). Therefore, fetuses with omphalocele/umbilical hernia, regardless of whether it is isolated or nonisolated, should trigger the consideration of BWS. Other major findings in fetal BWS include polyhydramnios (33.1%), macrosomia (32.5%), macroglossia (18.1%), and organomegaly (17.5%). Except for polyhydramnios, all major findings in fetal BWS are also common in postnatal cases. Macrosomia is known to be the most common features of BWS, presenting in about 80% of patients [61]. Nevertheless, causes of macrosomia are diverse and may relate to maternal conditions (e.g., diabetes, obesity), genetics, and fetal complications. Several overgrowth syndromes that show feature of macrosomia but are usually not associated with visceromegaly, macroglossia, or omphalocele should be differentiated from BWS, e.g., Simpson–Golabi–Behmel syndrome type 1 (OMIM 312870), an X-linked recessive disease caused by mutations in *GPC3* gene (OMIM *300037) on chromosome Xq26; Perlman syndrome (OMIM 267000), an autosomal recessive disease caused by mutations in *DIS3L2* gene (OMIM *614184) on chromosome 2q37; and Sotos syndrome (OMIM 117550), an autosomal dominant disease caused by mutations in *NSD1* gene (OMIM *117550) on chromosome 5q35 [36]. Molecular analyses, together with the ultrasonographic findings, contribute to the differential diagnosis. In the latest EUCID.net clinical scoring system, macrosomia is no longer considered a cardinal feature of BWS. In contrast, hemihyperplasia, a typical feature of BWS presenting in 30–35% of patients [61], was seldom reported in affected fetuses, suggesting a late-onset or prenatally indistinguishable condition.

Some genotype–phenotype correlations were identified in fetal BWS. Abdominal wall defects are dominant in fetuses with ICR2 hypomethylation. Macrosomia and organomegaly are more frequent in patients with ICR1 hypermethylation. The results indicated that different imprinted genes respond to different phenotypes [62,63]. However, it is interesting to note that not all fetal features associated with ICR1 hypermethylation and ICR2 hypomethylation were common in patUPD11p15.5, which theoretically causes ICR1 hypermethylation and ICR2 hypomethylation. For example, abdominal wall defects dominant in ICR2 hypomethylation were not common in patUPD11p15.5 (37.5% vs. 13.8%). Other molecular mechanisms, in addition to ICR1 hypermethylation and ICR2 hypomethylation, may play roles in the phenotypic responses of patUPD11p15.5.

A prenatal scoring system for fetal BWS diagnosis was recently proposed [49]. The system classified the prenatal findings into major, minor/suggestive, and supportive features and gave 3, 2, and 1 weighting points in order. Using a criterion of 3 points, however, only 58% (50/89) of prenatal cases were indicated for molecular confirmation [49]. The diagnostic rate by the abnormal scoring is not very high, mainly due to the broad phenotypic spectrum but also limited features manifested in fetal BWS that render many affected fetuses as not meeting the diagnostic criteria (Table S1). A practical approach combining clinical imaging and molecular modalities to facilitate the prenatal diagnosis of BWS is thus demanded. Given the finding of the genotype–phenotype correlation in fetuses as mentioned above as well as recent advances in molecular methodology, molecular tests could be considered if fetal BWS was not ruled out, and even only one typical feature was noted (Table S1 and Table 1). For example, abdominal wall defects are strong indications for methylation analysis of ICR2, and macrosomia and organomegaly are indicated for methylation analysis of ICR1. MS-MLPA is suggested as the first line of diagnostic test for fetuses without family history because it can simultaneously detect the ICR1/ICR2 methylation statuses and copy numbers. The test is not labor-intensive, and its cost is not too high (<USD 300 in Taiwan). MS-MLPA can solve the majority of the clinical cases in the prenatal scenario. In the 166 cases reviewed (Table S1), 89.8% (149/166; including 99 of ICR2 hypomethylation, 14 of ICR1 hypermethylation, 32 patUPD11p15.5, and 4 of chromosome deletions/duplications) can be confirmed the BWS diagnosis by MS-MLPA. Moreover, combining THE results of methylation and CNV analyses, the molecular mechanism of epigenetic and genetic aberrations may be inferred and even determined (Table S2). For example, detection of ICR2 hypomethylation, the most common causation of fetal BWS (52.0%), without CNV may indicate patUPD in ICR2 or simply methylation change, whereas ICR2 hypomethylation with CNV may suggest deletions in maternal ICR2 or duplications in paternal ICR2 depending on the CNV types detected (Table S2). Information of genotype–phenotype correlations (Table 1) also contributed to the molecular implications for prenatal cases. Following genetic tests (such as karyotype and/or fluorescence in situ hybridization (FISH) to identify translocation and single nucleotide polymorphism-CMA and/or short tandem repeat (STR) analysis to confirm patUPD) can be offered to clarify the underlying causes. For those fetuses or couples that showed positive familial history with a known molecular defect, prenatal tests or PGD are indicated according to the formats of genetic alternations.

In addition to fetal anomalies, ART have been linked to BWS. Children conceived by ART are at approximately 4–10-fold increased risk of BWS [5,64,65,66,67]. Two prenatal series included in our review showed that the ratios of BWS-affected pregnancies conceived by ART were 15.8% (3/19) [47] and 19.1% (17/89) [49]. A number of reports showed that ART disturbs the DNA methylation at imprinted loci, supporting the notion that ART may cause the imprinting disorders, including BWS [64,65,66,68]. Additionally, several case reports and case series studies showed that monozygotic twins, mostly female, are remarkably increased in frequency in BWS pregnancies [49,69,70]. Monozygotic twinning is well-known to be associated with hypomethylation in ICR2 [71]. A cohort study included in our review showed that the twinning rate is highest in the BWS subgroup of ICR2 hypomethylation (77.78%; 7/9) [49]. Because both ART and monozygotic twins are risk factors of BWS, prenatal screening of BWS by MS-MLPA in these case groups may be helpful for the early detection of disease. Nevertheless, since common complications in pregnancies with BWS fetuses (e.g., gestational hypertension, pre-eclampsia, gestational diabetes mellitus, vaginal bleeding) usually start after the GA of 22 weeks [15], screening of BWS at an early gestation when pregnancy complications yet occur may cause excess anxiety although BWS is extremely rare, and late TOP remains a feasible and legal rescuing option in Taiwan [72].

Finally, it should keep in mind that prenatal diagnosis of BWS remains a challenge because the range of molecular disturbances cannot be expected, and mosaicism may occur and change over time during the pregnancy. Moreover, it has been shown that MS-MLPA on CVS might not reflect the actual epigenetic constitution of the fetus because the loci of interest might not have reached its final imprinting status in CVS, and the rate at which the imprinting is set may differ between and even within loci [73]. These factors can affect the reliability of prenatal test for BWS. TOP was commonly opted for parents with a BWS pregnancy. However, treatments are currently available for some of the symptoms of BWS, and BWS features may become unapparent with age, making BWS not always a terrible condition [2,74]. Therefore, prior to offering prenatal diagnosis for BWS, a detailed counseling concerning the diagnostic procedures, technological limitations of testing (e.g., MS-MLPA on CVS), and ethical issues involved should be provided. People accepting a molecular diagnosis of fetal BWS should be also informed that a normal result does not completely exclude the diagnosis.

## 5. Conclusions

Molecular tests are suggested for fetuses if BWS was not ruled out, even only one typical feature was noted due to the broad phenotypic spectrum but limited features manifested in affected fetuses. MS-MLPA is considered as the first line of diagnostic test because it solves the majority of the clinical BWS cases in the prenatal scenario. Subsequent genetic tests (e.g., CMA, STR analyses, DNA sequencing) can be offered if indicated to full delineation of molecular pathologies. By a comprehensive understanding of the molecular pathologies underlying BWS spectrum and utilizing the appropriate molecular tools, prenatal diagnosis of BWS is possible. However, prenatal counseling including the diagnostic procedures, technological limitations of testing, and ethical issues is necessary.

## Figures and Tables

**Table 1:** Summary of the correlation of the prenatal features and genotypes of 166 reported BWS patients (see Table S1 for detail).

	ICR2 Hypomethylation ^†^	ICR1 Hypermethylation ^†^	patUPD11p15.5 ^†^	Others ^†, ‡^	Total ^†^
Number of cases	99	14	32	21	166
Abdominal wall defects (e.g., umbilical hernia/omphalocele)	57 (57.6%)	0 (0)	6 (18.8%)	8 (38.1%)	71 (42.8%)
Macroglossia	20 (20.2%)	6 (42.9%)	2 (6.3%)	2 (9.5%)	30 (18.1%)
Macrosomia	26 (26.3%)	7 (50.0%)	11 (34.4%)	10 (47.6%)	54 (32.5%)
Organomegaly (e.g., nephromegaly, hepatomegaly, cardiomegaly)	14 (14.1%)	8 (57.1%)	6 (18.8%)	4 (19.3%)	29 (17.5%)
Polyhydramnios	33 (33.3%)	7 (50.0%)	7 (21.9%)	8 (38.1%)	55 (33.1%)
Placentomegaly	9 (9.1%)	0 (0)	2 (6.3%)	2 (9.5%)	13 (7.8%)
Corpus callosum anomaly	1 (1.0%)	0 (0)	1 (3.1%)	0 (0)	2 (1.2%)
Tumor (e.g., placental tumor, macroglossia, intracardiac rhabdomyoma)	1 (1.0%)	3 (21.4%)	1 (3.1%)	1 (4.8%)	6 (3.6%)

^†^ Numbers in the parentheses indicate the percentage of case number. ^‡^ Six cases without genetic data; six cases with a negative result of genetic analysis; five cases with chromosomal 11p15 abnormalities (including deletions, duplications, and rearrangements); four cases with *CDKN1C* mutations. ICR1, imprinting control region 1; ICR2, imprinting control region 2; patUPD, paternal uniparental disomy.

## Data Availability

Data are available upon reasonable request.

## Supplementary Materials

The following supporting information can be downloaded at: https://www.mdpi.com/article/10.3390/diagnostics12071709/s1, Table S1: Prenatal ultrasound finding, genetic diagnosis, imprinting control region 1 (ICR1)/ICR2 methylation status, timing of diagnosis, and outcomes of 166 reported patients with Beckwith–Wiedemann syndrome (BWS); Table S2: Common BWS molecular pathologies can be inferred from methylation-specific multiplex ligation-dependent probe amplification (MS-MLPA) of chromosome 11p15 and analyzed by subsequent genetic tests.

## References

[1] Williams D.H., Gauthier D.W., Maizels M. (2005). Prenatal diagnosis of Beckwith-Wiedemann syndrome. Prenat. Diagn..

[2] Brioude F., Kalish J.M., Mussa A., Foster A.C., Bliek J., Ferrero G.B., Boonen S.E., Cole T., Baker R., Bertoletti M. (2018). Expert consensus document: Clinical and molecular diagnosis, screening and management of Beckwith-Wiedemann syndrome: An international consensus statement. Nat. Rev. Endocrinol..

[3] Mussa A., Molinatto C., Baldassarre G., Riberi E., Russo S., Larizza L., Riccio A., Ferrero G.B. (2016). Cancer Risk in Beckwith-Wiedemann Syndrome: A Systematic Review and Meta-Analysis Outlining a Novel (Epi)Genotype Specific Histotype Targeted Screening Protocol. J. Pediatr..

[4] Mussa A., Russo S., De Crescenzo A., Chiesa N., Molinatto C., Selicorni A., Richiardi L., Larizza L., Silengo M.C., Riccio A. (2013). Prevalence of Beckwith-Wiedemann syndrome in North West of Italy. Am. J. Med. Genet. A.

[5] Mussa A., Molinatto C., Cerrato F., Palumbo O., Carella M., Baldassarre G., Carli D., Peris C., Riccio A., Ferrero G.B. (2017). Assisted Reproductive Techniques and Risk of Beckwith-Wiedemann Syndrome. Pediatrics.

[6] Lustig O., Ariel I., Ilan J., Lev-Lehman E., De-Groot N., Hochberg A. (1994). Expression of the imprinted gene H19 in the human fetus. Mol. Reprod. Dev..

[7] Zheng J.F., Guo N.H., Zi F.M., Cheng J. (2020). Long Noncoding RNA H19 Promotes Tumorigenesis of Multiple Myeloma by Activating BRD4 Signaling by Targeting MicroRNA 152-3p. Mol. Cell Biol..

[8] Lee J.E., Pintar J., Efstratiadis A. (1990). Pattern of the insulin-like growth factor II gene expression during early mouse embryogenesis. Development.

[9] Matsuoka S., Edwards M.C., Bai C., Parker S., Zhang P., Baldini A., Harper J.W., Elledge S.J. (1995). p57KIP2, a structurally distinct member of the p21CIP1 Cdk inhibitor family, is a candidate tumor suppressor gene. Genes Dev..

[10] Jespersen T., Grunnet M., Olesen S.P. (2005). The KCNQ1 potassium channel: From gene to physiological function. Physiology.

[11] Mohammad F., Mondal T., Guseva N., Pandey G.K., Kanduri C. (2010). Kcnq1ot1 noncoding RNA mediates transcriptional gene silencing by interacting with Dnmt1. Development.

[12] Niemitz E.L., DeBaun M.R., Fallon J., Murakami K., Kugoh H., Oshimura M., Feinberg A.P. (2004). Microdeletion of LIT1 in familial Beckwith-Wiedemann syndrome. Am. J. Hum. Genet..

[13] Sparago A., Cerrato F., Vernucci M., Ferrero G.B., Silengo M.C., Riccio A. (2004). Microdeletions in the human H19 DMR result in loss of IGF2 imprinting and Beckwith-Wiedemann syndrome. Nat. Genet..

[14] Choufani S., Shuman C., Weksberg R. (2013). Molecular findings in Beckwith-Wiedemann syndrome. Am. J. Med. Genet. C Semin. Med. Genet..

[15] Borjas Mendoza P.A., Mendez M.D. (2022). Beckwith Wiedemann Syndrome. StatPearls [Internet].

[16] Elliott M., Bayly R., Cole T., Temple I.K., Maher E.R. (1994). Clinical features and natural history of Beckwith-Wiedemann syndrome: Presentation of 74 new cases. Clin. Genet..

[17] DeBaun M.R., Tucker M.A. (1998). Risk of cancer during the first four years of life in children from The Beckwith-Wiedemann Syndrome Registry. J. Pediatr..

[18] Shieh H.F., Estroff J.A., Barnewolt C.E., Zurakowski D., Tan W.H., Buchmiller T.L. (2019). Prenatal imaging throughout gestation in Beckwith-Wiedemann syndrome. Prenat. Diagn..

[19] Shuman C., Beckwith J.B., Weksberg R., Adam M.P., Ardinger H.H., Pagon R.A., Wallace S.E., Bean L.J.H., Gripp K.W., Mirzaa G.M., Amemiya A. (2019). Beckwith-Wiedemann Syndrome. 2000 Mar 3 [Updated 2016 Aug 11]. GeneReviews^®^ [Internet].

[20] Van Vuuren S.H., Damen-Elias H.A., Stigter R.H., van der Doef R., Goldschmeding R., de Jong T.P., Westers P., Visser G.H., Pistorius L.R. (2012). Size and volume charts of fetal kidney, renal pelvis and adrenal gland. Ultrasound Obstet. Gynecol..

[21] Hamada H., Fujiki Y., Obata-Yasuoka M., Watanabe H., Yamada N., Kubo T. (2001). Prenatal sonographic diagnosis of Beckwith-Wiedemann syndrome in association with a single umbilical artery. J. Clin. Ultrasound.

[22] Pelizzo G., Conoscenti G., Kalache K.D., Vesce F., Guerrini P., Cavazzini L. (2003). Antenatal manifestation of congenital pancreatoblastoma in a fetus with Beckwith-Wiedemann syndrome. Prenat. Diagn..

[23] Le Caignec C., Gicquel C., Gubler M.C., Guyot C., You M.C., Laurent A., Joubert M., Winer N., David A., Rival J.M. (2004). Sonographic findings in Beckwith-Wiedemann syndrome related to H19 hypermethylation. Prenat. Diagn..

[24] Mulik V., Wellesley D., Sawdy R., Howe D.T. (2004). Unusual prenatal presentation of Beckwith-Wiedemann syndrome. Prenat. Diagn..

[25] Sinico M., Touboul C., Haddad B., Encha-Razavi F., Paniel J.B., Gicquel C., Gérard-Blanluet M. (2004). Giant omphalocele and "prune belly" sequence as components of the Beckwith-Wiedemann syndrome. Am. J. Med. Genet. Part A.

[26] Gocmen R., Basaran C., Karcaaltincaba M., Cinar A., Yurdakok M., Akata D., Haliloglu M. (2005). Bilateral hemorrhagic adrenal cysts in an incomplete form of Beckwith-Wiedemann syndrome: MRI and prenatal US findings. Abdom. Imaging.

[27] Aagaard-Tillery K.M., Buchbinder A., Boente M.P., Ramin K.D. (2007). Beckwith-Wiedemann syndrome presenting with an elevated triple screen in the second trimester of pregnancy. Fetal Diagn. Ther..

[28] Grati F.R., Turolla L., D’Ajello P., Ruggeri A., Miozzo M., Bracalente G., Baldo D., Laurino L., Boldorini R., Frate E. (2007). Chromosome 11 segmental paternal isodisomy in amniocytes from two fetuses with omphalocoele: New highlights on phenotype-genotype correlations in Beckwith-Wiedemann syndrome. J. Med. Genet..

[29] Gomes M.V., Gomes C.C., Pinto W., Ramos E.S. (2007). Methylation pattern at the KvDMR in a child with Beckwith-Wiedemann syndrome conceived by ICSI. Am. J. Med. Genet. A.

[30] Ma G.C., Chang S.D., Chang Y., Chang S.P., Yang C.W., Lee M.J., Lee T.H., Chen M. (2008). Rapid prenatal confirmation of LIT1 hypomethylation using a novel quantitative method (E-Q-PCR) in fetuses with Beckwith-Wiedemann syndrome impressed with ultrasonography. Fertil. Steril..

[31] Percesepe A., Bertucci E., Ferrari P., Lugli L., Ferrari F., Mazza V., Forabosco A. (2008). Familial Beckwith-Wiedemann syndrome due to CDKN1C mutation manifesting with recurring omphalocele. Prenat. Diagn..

[32] Descartes M., Romp R., Franklin J., Biggio J.R., Zehnbauer B. (2008). Constitutional H19 hypermethylation in a patient with isolated cardiac tumor. Am. J. Med. Genet. Part A.

[33] Ramadan G.I., Kennea N.L. (2009). Beckwith-Wiedemann syndrome associated with congenital hypothyroidism in a preterm neonate: A case report and literature review. J. Perinatol..

[34] Bui C., Picone O., Mas A.E., Levaillant J.M., Ami O., Netchine I., Frydman R., Senat M.V. (2009). Beckwith-Wiedemann syndrome in association with posterior hypoplasia of the cerebellar vermis. Prenat. Diagn..

[35] Sorrentino S., Conte M., Nozza P., Granata C., Capra V., Avanzini S., Garaventa A. (2010). Simultaneous occurrence of pancreatoblastoma and neuroblastoma in a newborn with Beckwith-Wiedemann syndrome. J. Pediatr. Hematol. Oncol..

[36] Storm D.W., Hirselj D.A., Rink B., O’Shaughnessy R., Alpert S.A. (2011). The prenatal diagnosis of Beckwith-Wiedemann syndrome using ultrasound and magnetic resonance imaging. Urology.

[37] Aoki A., Shiozaki A., Sameshima A., Higashimoto K., Soejima H., Saito S. (2011). Beckwith-Wiedemann syndrome with placental chorangioma due to H19-differentially methylated region hypermethylation: A case report. J. Obstet. Gynaecol. Res..

[38] Eckmann-Scholz C., Jonat W. (2011). 3-D ultrasound imaging of a prenatally diagnosed Beckwith-Wiedemann syndrome. Arch. Gynecol. Obstet..

[39] Guanciali-Franchi P., Di Luzio L., Iezzi I., Celentano C., Matarrelli B., Liberati M., Palka G. (2012). Elevated maternal serum α-fetoprotein level in a fetus with Beckwith-Wiedemann syndrome in the second trimester of pregnancy. J. Prenat. Med..

[40] Moreira-Pinto J., Pereira J., Osório A., Enes C., Mota C.R. (2012). Beckwith-Wiedemann syndrome, delayed abdominal wall closure, and neonatal intussusception--case report and literature review. Fetal. Pediatr. Pathol..

[41] Longardt A.C., Nonnenmacher A., Graul-Neumann L., Opgen-Rhein B., Henrich W., Bührer C., Hüseman D. (2014). Fetal intracardiac rhabdomyoma in Beckwith-Wiedemann syndrome. J. Clin. Ultrasound.

[42] Chen C.P., Su Y.N., Chen S.U., Chang T.Y., Wu P.C., Chern S.R., Wu P.S., Kuo Y.L., Wang W. (2014). Prenatal diagnosis of hypomethylation at KvDMR1 and Beckwith-Wiedemann syndrome in a pregnancy conceived by intracytoplasmic sperm injection and in vitro fertilization and embryo transfer. Taiwan. J. Obstet. Gynecol..

[43] Jurkiewicz D., Kugaudo M., Tańska A., Wawrzkiewicz-Witkowska A., Tomaszewska A., Kucharczyk M., Cieślikowska A., Ciara E., Krajewska-Walasek M. (2015). 11p15 duplication and 13q34 deletion with Beckwith-Wiedemann syndrome and factor VII deficiency. Pediatr. Int..

[44] Chen K.J., Liu Y.M., Li C.H., Chang Y.L., Chang S.D. (2016). Prenatal diagnosis of paternal duplication of 11p15.5→14.3, Its implication of Beckwith-Wiedemann syndrome. Taiwan. J. Obstet. Gynecol..

[45] Wang Q., Geng Q., Zhou Q., Luo F., Li P., Xie J. (2017). De novo paternal origin duplication of chromosome 11p15.5, report of two Chinese cases with Beckwith-Wiedemann syndrome. Mol. Cytogenet..

[46] Beaufrère A., Bonnière M., Tantau J., Roth P., Schaerer E., Brioude F., Netchine I., Bessières B., Gelot A., Vekemans M. (2018). Corpus Callosum Abnormalities and Short Femurs in Beckwith-Wiedemann Syndrome: A Report of Two Fetal Cases. Fetal Pediatr. Pathol..

[47] Abbasi N., Moore A., Chiu P., Ryan G., Weksberg R., Shuman C., Steele L., Chitayat D. (2021). Prenatally diagnosed omphaloceles: Report of 92 cases and association with Beckwith-Wiedemann syndrome. Prenat. Diagn..

[48] Baker S.W., Ryan E., Kalish J.M., Ganguly A. (2021). Prenatal molecular testing and diagnosis of Beckwith-Wiedemann syndrome. Prenat. Diagn..

[49] Carli D., Bertola C., Cardaropoli S., Ciuffreda V.P., Pieretto M., Ferrero G.B., Mussa A. (2021). Prenatal features in Beckwith-Wiedemann syndrome and indications for prenatal testing. J. Med. Genet..

[50] Wang K.H., Kupa J., Duffy K.A., Kalish J.M. (2020). Diagnosis and Management of Beckwith-Wiedemann Syndrome. Front. Pediatr..

[51] Queremel Milani D.A., Chauhan P.R. (2022). Genetics, Mosaicism. StatPearls [Internet].

[52] Moutou C., Junien C., Henry I., Bonaïti-Pellié C. (1992). Beckwith-Wiedemann syndrome: A demonstration of the mechanisms responsible for the excess of transmitting females. J. Med. Genet..

[53] Evans M.I., Evans S.M., Bennett T.A., Wapner R.J. (2018). The price of abandoning diagnostic testing for cell-free DNA screening. Prenat. Diagn..

[54] Evans M.I., Andrioles S., Curtis J., Evans S.M., Kessler A.A., Rubenstein A.F. (2018). The epidemic of abnormal copy number variant cases missed because of reliance upon noninvasive prenatal screening. Prenat. Diagn..

[55] Benn P., Cuckle H., Pergament E. (2013). Non-invasive prenatal testing for aneuploidy: Current status and future prospects. Ultrasound Obstet. Gynecol..

[56] Cheng H.H., Ma G.C., Tsai C.C., Wu W.J., Lan K.C., Hsu T.Y., Yang C.W., Chen M. (2016). Confined placental mosaicism of double trisomies 9 and 21: Discrepancy between non-invasive prenatal testing, chorionic villus sampling and postnatal confirmation. Ultrasound. Obstet. Gynecol..

[57] Liao C.H., Chang M.Y., Ma G.C., Chang S.P., Lin C.F., Lin W.H., Chen H.F., Chen S.U., Lee Y.C., Chao C.C. (2019). Preimplantation Genetic Diagnosis of Neurodegenerative Diseases: Review of Methodologies and Report of Our Experience as a Regional Reference Laboratory. Diagnostics.

[58] Yang I.J., Tu Y.A., Pan S.P., Huang T.C., Chen C.L., Lin M.W., Tsai Y.Y., Yao Y.L., Su Y.N., Chen S.U. (2022). First report of a successful pregnancy by preimplantation genetic testing for Beckwith-Wiedemann syndrome. Taiwan. J. Obstet. Gynecol..

[59] Adams A.D., Stover S., Rac M.W. (2021). Omphalocele-What should we tell the prospective parents?. Prenat. Diagn..

[60] Van den Veyver I.B. (2021). Improving the prenatal diagnosis of Beckwith-Wiedemann syndrome. Prenat. Diagn..

[61] Gicquel C., Rossignol S., Le Bouc Y. (2005). Beckwith-Wiedemann Syndrome. Orphanet. Encyclopedia.

[62] DeBaun M.R., Niemitz E.L., McNeil D.E., Brandenburg S.A., Lee M.P., Feinberg A.P. (2002). Epigenetic alterations of H19 and LIT1 distinguish patients with Beckwith-Wiedemann syndrome with cancer and birth defects. Am. J. Hum. Genet..

[63] Rump P., Zeegers M.P., van Essen A.J. (2005). Tumor risk in Beckwith-Wiedemann syndrome: A review and meta-analysis. Am. J. Med. Genet. Part A.

[64] DeBaun M.R., Niemitz E.L., Feinberg A.P. (2003). Association of in vitro fertilization with Beckwith-Wiedemann syndrome and epigenetic alterations of LIT1 and H19. Am. J. Hum. Genet..

[65] Gicquel C., Gaston V., Mandelbaum J., Siffroi J.P., Flahault A., Le Bouc Y. (2003). In vitro fertilization may increase the risk of Beckwith-Wiedemann syndrome related to the abnormal imprinting of the KCN1OT gene. Am. J. Hum. Genet..

[66] Maher E.R., Brueton L.A., Bowdin S.C., Luharia A., Cooper W., Cole T.R., Macdonald F., Sampson J.R., Barratt C.L., Reik W. (2003). Beckwith-Wiedemann syndrome and assisted reproduction technology (ART). J. Med. Genet..

[67] Johnson J.P., Beischel L., Schwanke C., Styren K., Crunk A., Schoof J., Elias A.F. (2018). Overrepresentation of pregnancies conceived by artificial reproductive technology in prenatally identified fetuses with Beckwith-Wiedemann syndrome. J. Assist. Reprod. Genet..

[68] Halliday J., Oke K., Breheny S., Algar E., Amor D.J. (2004). Beckwith-Wiedemann syndrome and IVF: A case-control study. Am. J. Hum. Genet..

[69] Weksberg R., Shuman C., Caluseriu O., Smith A.C., Fei Y.L., Nishikawa J., Stockley T.L., Best L., Chitayat D., Olney A. (2002). Discordant KCNQ1OT1 imprinting in sets of monozygotic twins discordant for Beckwith-Wiedemann syndrome. Hum. Mol. Genet..

[70] Fontana L., Bedeschi M.F., Cagnoli G.A., Costanza J., Persico N., Gangi S., Porro M., Ajmone P.F., Colapietro P., Santaniello C. (2020). (Epi)genetic profiling of extraembryonic and postnatal tissues from female monozygotic twins discordant for Beckwith-Wiedemann syndrome. Mol. Genet. Genomic. Med..

[71] Cohen J.L., Duffy K.A., Sajorda B.J., Hathaway E.R., Gonzalez-Gandolfi C.X., Richards-Yutz J., Gunter A.T., Ganguly A., Kaplan J., Deardorff M.A. (2019). Diagnosis and management of the phenotypic spectrum of twins with Beckwith-Wiedemann syndrome. Am. J. Med. Genet. A.

[72] Chen C.H., Hsieh H.C., Tsai H.D., Chen T.H., Chen M. (2009). Cardiac tamponade: An alternative procedure for late feticide. Taiwan. J. Obstet. Gynecol..

[73] Paganini L., Carlessi N., Fontana L., Silipigni R., Motta S., Fiori S., Guerneri S., Lalatta F., Cereda A., Sirchia S. (2015). Beckwith-Wiedemann syndrome prenatal diagnosis by methylation analysis in chorionic villi. Epigenetics.

[74] Mussa A., Di Candia S., Russo S., Catania S., De Pellegrin M., Di Luzio L., Ferrari M., Tortora C., Meazzini M.C., Brusati R. (2016). Recommendations of the Scientific Committee of the Italian Beckwith-Wiedemann Syndrome Association on the diagnosis, management and follow-up of the syndrome. Eur. J. Med. Genet..

