# Supplementary material for: Proposal for Practical Approach in Prenatal Diagnosis of Beckwith–Wiedemann Syndrome and Review of the Literature
Source: Diagnostics (Basel). 2022 Jul 13;12(7):1709. doi: 10.3390/diagnostics12071709 (PMC9315620; doi:10.3390/diagnostics12071709)
Supplement: Supplementary file 1 [file diagnostics-12-01709-s001.zip › Table S1.pdf]

Table S1. Prenatal ultrasound finding, genetic diagnosis, imprinting control region 1 (ICR1)/ICR2 methylation status, timing of diagnosis, and outcomes of 166 reported patients with Beckwith–Wiedemann Syndrome (BWS).

Case no.	Prenatal ultrasound finding*	Genetic diagnosis	ICR1/ICR2 methylation status†	Timing of diagnosis/outcomes	Reference
1	Umbilical hernia	Normal aCGH	ICR2 hypomethylation	Post/Live birth	This study (Patient 1)
2	Omphalocele	Normal aCGH	ICR2 hypomethylation	Pre/TOP	This study (Patient 2)
3	Nephromegaly, macroglossia	arr[GRCh37] 11p15.5(1996741_2028877)×1 mat	ICR1 hypermethylation	Pre/TOP	This study (Patient 3)
4	Omphalocele, corpus callosum hypoplasia, short femurs	NR	ICR2 hypomethylation	Pre/TOP	Beaufrère et al. (2018) (Patient 1) [46]
5	Nuchal hypertranslucency, intrauterine growth restriction, omphalocele, feet deformity, facial dysmorphism, short corpus callosum	patUPD11p15.5	ICR1 hypermethylation; ICR2 hypomethylation	Pre/TOP	Beaufrère et al. (2018) (Patient 2) [46]
6	Protruding tongue, nephromegaly, hepatomegaly, polyhydramnios, macrosomia	arr[GRCh37] 11p15.5(1632167_2527910)×3	ICR1 hypermethylation	Pre/TOP	Wang et al. (2017) (Patient 1) [45]
7	Left-sided congenital diaphragmatic hernia, polyhydraminos, macrosomia	arr[GRCh37] 11p15.5p14.3(196966_250338960)×3 dn, 11q24.3q25(9129510272_134868407)×1	[ICR1 hypermethylation; ICR2 hypomethylation]	Pre/TOP	Chen et al. (2016) [44]
8	Polyhydramnios, macrosomia, hepatomegaly	arr[GRCh37] 11p15.5p15.4(205983_4920324)×3 dn, 13q34(112350047_115091736)×1 dn	ICR1 hypermethylation; ICR2 hypomethylation	Pre/Live birth	Jurkiewicz et al. (2015) [43]
9	Omphalocele	Normal aCGH	ICR2 hypomethylation	Pre/NR	Chen et al. (2014) [42]
10	Intracardiac rhabdomyoma, polyhydramnios	NR	ICR1 hypermethylation; ICR2 hypomethylation	Post/Live birth	Longardt et al. (2014) [41]
11	Omphalocele	NR	ICR2 hypomethylation	Post/Live birth	Moreira-Pinto et al. (2012) [40]
12	Macroglossia, macrosomia, hepatomegaly, nephromegaly, cardiomegaly, polyhydramnios	NR	ICR1 hypermethylation	Post/Live birth	Guanciali-Franchi et al. (2012) [39]
13	Hydropic placenta, hepatomegaly, polyhydramnios, macrosomia, macroglossia	NR	ICR2 hypomethylation	Post/Live birth	Eckmann-Scholz et al. (2011) [38]
14	Placental tumor, polyhydramnios, macroglossia, hepatomegaly, nephromegaly	patUPD11p15.5 and *CDKN1C* mutation were ruled out	ICR1 hypermethylation	Pre/Live birth	Aoki et al. (2011) [37]
15	Nephromegaly, macroglossia	patUPD11p15.5	[ICR1 hypermethylation; ICR2 hypomethylation]	Pre/Live birth	Storm et al. (2011) [36]
16	Mesenteric cystic lesion	Mosaic patUPD11p15.5	[ICR1 hypermethylation; ICR2 hypomethylation]	Post/Live birth	Sorrentino et al. (2010) [35]
17	Omphalocele, open of the fourth ventricle, hypoplasia of the posterior vermis	NR	ICR2 hypomethylation	Post/Live birth	Bui et al. (2009) [34]
18	Omphalocele	NR	ICR2 hypomethylation	Post/Live birth	Ramadan et al. (2009) [33]
19	Intracardiac tumor (right atrial mass)	NR	ICR1 hypermethylation	Post/Live birth	Descartes et al. (2008) [32]
20	Omphalocele, macrosomia	*CDKN1C* mutation	NR	Pre/Live birth	Percesepe et al. (2008) [31]

21	Macrosomia, macroglossia, omphalocele, polyhydramnios, abdominal distention	NR	ICR2 hypomethylation	Pre/TOP	Ma et al. (2008) (Patient 1) [30]
22	Macrosomia, macroglossia, polyhydramnios, cardiomegaly, nephromegaly	NR	ICR2 hypomethylation	Pre/TOP	Ma et al. (2008) (Patient 2) [30]
23	Omphalocele, polyhydramnios, macrosomia, macroglossia, nephromegaly	NR	ICR2 hypomethylation	Post/Live birth	Gomes et al. (2007) [29]
24	Omphalocele	Mosaic patUPD11p15.5	[ICR1 hypermethylation; ICR2 hypomethylation]	Pre/TOP	Grati et al. (2007) (Patient 1) [28]
25	Omphalocele	Mosaic patUPD11p15.5	[ICR1 hypermethylation; ICR2 hypomethylation]	Pre/TOP	Grati et al. (2007) (Patient 2) [28]
26	Two-vessel cord, enlarged right atrium, foreshortened femurs, echogenic bowel	NR	ICR1 hypermethylation	Post/Live birth	Aagaard-Tillery et al. (2007) [27]
27	Renal cystic lesion	NR	NR	Post/Live birth	Gocmen et al. (2005) [26]
28	Omphalocele, polyhydramnios, nephromegaly	NR	NR	Pre/TOP	Williams et al. (2005) (Patient 1) [1]
29	Omphalocele, hepatomegaly, nephromegaly, macroglossia	NR	NR	Post/Live birth	Williams et al. (2005) (Patient 2) [1]
30	Omphalocele, megacystis, ureteric dilatation, hydronephrosis	NR	ICR2 hypomethylation	Pre/TOP	Sinico et al. (2004) [25]
31	Enlarged placenta with cystic lesion, macrosomia, hepatomegaly, enlarged pancreas, multiple small cystic areas in placenta	Mosaic patUPD11p15.5 or paternal duplication of 11p15.5	[ICR1 hypermethylation; ICR2 hypomethylation]	Pre/TOP	Mulik et al. (2004) [24]
32	Nephromegaly, biparietal diameter, head circumference, and abdominal circumference were increased for gestational age	NR	ICR1 hypermethylation	Pre/TOP	Le Caignec et al. (2004) [23]
33	Intra-abdominal cyst, polyhydramnios	patUPD11p15.5	[ICR1 hypermethylation; ICR2 hypomethylation]	Post/Live birth	Pelizzo et al. (2003) [22]
34	Omphalocele, single umbilical artery, polyhydraminos, macrosomia	NR	NR	Pre/Live birth	Hamada et al. (2001) [21]
35	Omphalocele,Dandy-Walker malformation, macroglossia, polyhydramnios, nephromegaly	NR	ICR2 hypomethylation	Post/Live birth	Abbasi et al. (2021) (Patient 1) [47]
36	Omphalocele	NR	ICR2 hypomethylation	Post/Live birth	Abbasi et al. (2021) (Patient 2) [47]
37	Omphalocele,polyhydramnios, placentomegaly	NR	ICR2 hypomethylation	Pre/Live birth	Abbasi et al. (2021) (Patient 3) [47]
38	Omphalocele	NR	ICR2 hypomethylation	Post/Live birth	Abbasi et al. (2021) (Patient 4) [47]
39	Omphalocele, macrosomia, polyhydramnios	NR	ICR2 hypomethylation	Pre/Live birth	Abbasi et al. (2021) (Patient 5) [47]

40	Omphalocele, macrosomia, polyhydramnios	NR	ICR2 hypomethylation	Pre/Live birth	Abbasi et al. (2021) (Patient 6) [47]
41	Omphalocele, macrosomia, macroglossia, polyhydramnios	NR	ICR2 hypomethylation	Pre/Live birth	Abbasi et al. (2021) (Patient 7) [47]
42	Omphalocele	NR	ICR2 hypomethylation	Pre/Live birth	Abbasi et al. (2021) (Patient 8) [47]
43	Omphalocele, macroglossia, macrosomia, nephromegaly	NR	ICR2 hypomethylation	Pre/Live birth	Abbasi et al. (2021) (Patient 9) [47]
44	Omphalocele, Dandy-Walker malformation, echogenic bowel, liver calcifications, short long bones, punctate epiphyses, syndactyly, placentomegaly, polyhydramnios, fetal growth restriction	11p15.4 deletion including *CDKN1C*	NR	Pre/Live birth	Abbasi et al. (2021) (Patient 10) [47]
45	Omphalocele, polyhydramnios	NR	ICR2 hypomethylation	Pre/Live birth	Abbasi et al. (2021) (Patient 11) [47]
46	Omphalocele, macrosomia, polyhydramnios	NR	ICR2 hypomethylation	Pre/Live birth	Abbasi et al. (2021) (Patient 12) [47]
47	Omphalocele, short cervix	NR	ICR2 hypomethylation	Post/Live birth	Abbasi et al. (2021) (Patient 13) [47]
48	Omphalocele	NR	ICR2 hypomethylation	Pre/Live birth	Abbasi et al. (2021) (Patient 14) [44]
49	Omphalocele, macrosomia, polyhydramnios, nephromegaly	NR	ICR2 hypomethylation	Pre/Live birth	Abbasi et al. (2021) (Patient 5) [47]
50	Omphalocele, macrosomia, polyhydramnios	NR	ICR2 hypomethylation	Post/Live birth	Abbasi et al. (2021) (Patient 16) [47]
51	Omphalocele, macrosomia, polyhydramnios	NR	ICR2 hypomethylation	Pre/*In utero* demise	Abbasi et al. (2021) (Patient 17) [47]
52	Omphalocele, cystic hygroma	NR	ICR2 hypomethylation	Post/Live birth	Abbasi et al. (2021) (Patient 18) [47]
53	Omphalocele, polyhydramnios, macroglossia	NR	ICR2 hypomethylation	Post/Live birth	Abbasi et al. (2021) (Patient 19) [47]
54-70	Omphalocele (15), macroglossia (5), placentomegaly (5), plancental mesenchymal dysplasia (0), organomegaly (7), polyhydramnios (1), macrosomia (1)	NR	ICR2 hypomethylation	Pre/NR	Baker et al. (2021) (17 patients) [48]
71	Omphalocele (0), macroglossia (0), placentomegaly (0), plancental mesenchymal dysplasia (0), organomegaly (1), polyhydramnios (1), macrosomia (0)	NR	ICR1 hypermethylation	Pre/NR	Baker et al. (2021) (1 patient) [48]
72-74	Omphalocele (2), macroglossia (1), placentomegaly (0), plancental mesenchymal dysplasia (0), organomegaly (1), polyhydramnios (0), macrosomia (0)	patUPD11p15.5	[ICR1 hypermethylation; ICR2 hypomethylation]	Pre/NR	Baker et al. (2021) (3 patients) [48]

75-77	Omphalocele (3), macroglossia (0), placentomegaly (1), plancental mesenchymal dysplasia (0), organomegaly (0), polyhydramnios (0), macrosomia (1)	*CDKN1C* mutations	NR	Pre/NR	Baker et al. (2021) (3 patients) [48]
78-129	Macroglossia (7), omphalocele (14), macrosomia (13), organomegaly (0), polyhydramnios (17), placentomegaly (2), tumor (0)	NR	ICR2 hypomethylation	Post/Live birth	Carli et al. (2021) (52 patients) [49]
130-135	Macroglossia (2), omphalocele (0), macrosomia (5), organomegaly (2), polyhydramnios (2), placentomegaly (0), tumor (0)	NR	ICR1 hypermethylation	Post/Live birth	Carli et al. (2021) (6 patients) [49]
136-157	Macroglossia (1), omphalocele (1), macrosomia (9), organomegaly (3), polyhydramnios (7), placentomegaly (0), tumor (1)	patUPD11p15.5	[ICR1 hypermethylation; ICR2 hypomethylation]	Post/Live birth	Carli et al. (2021) (22 patients) [49]
158-166	Macroglossia (1), omphalocele (0), macrosomia (5), organomegaly (1), polyhydramnios (2), placentomegaly (0), tumor (0)	1. 1 case: der(Y)t(Y;11)(p11.32-;p15.4+)<br>2. 2 cases: genetic analysis was not performed<br>3. 6 cases: negative result	NR	Post/Live birth	Carli et al. (2021) (9 patients) [49]

*Numbers in the parentheses indicate the number of cases.

†Methylation statuses in the square brackets are inferred from the results of molecular diagnosis.

patUPD: paternal uniparental disomy; GRCh37: genome reference consortium human build 37; mat: maternal origin; dn: de novo; Pre: prenatally; Post: postnatally; TOP: termination of pregnancy; NR: not reported.
