# Supplementary material for: Proposal for Practical Approach in Prenatal Diagnosis of Beckwith–Wiedemann Syndrome and Review of the Literature
Source: Diagnostics (Basel). 2022 Jul 13;12(7):1709. doi: 10.3390/diagnostics12071709 (PMC9315620; doi:10.3390/diagnostics12071709)
Supplement: Supplementary file 1 [file diagnostics-12-01709-s001.zip › Table S2.pdf]

Table S2. Common BWS molecular pathologies can be inferred from methylation-specific multiplex ligation-dependent probe amplification (MS-MLPA) of chromosome 11p15 and analyzed by subsequent genetic tests.

Pathogeny	MS-MLPA				Implications	Following genetic tests
	Methylation		CNV			
	ICR1	ICR2	ICR1	ICR2		
ICR2 hypomethylation	Normal	Hypomethylation	No change	No change	1. patUPD in ICR2<br>2. Simply methylation change	SNP-CMA, MS PCR, STR analysis
			No change	Deletion	Deletion in maternal ICR2	CMA/SNP-CMA, karyotype, FISH
			No change	Duplication	Duplication in paternal ICR2	CMA/SNP-CMA, karyotype, FISH
ICR1 hypermethylation	Hypermethylation	Normal	No change	No change	1. patUPD in ICR1<br>2. Simply methylation change	SNP-CMA, MS PCR, STR
			Deletion	No change	Deletion in maternal ICR1	CMA/SNP-CMA, karyotype, FISH
			Duplication	No change	Duplication in paternal ICR1	CMA/SNP-CMA, karyotype, FISH
patUPD11p15.5	Hypermethylation	Hypomethylation	No change	No change	patUPD11	SNP-CMA, STR analysis
Others	Normal	Normal	Normal	Normal	1. Mutations in BWS-related genes (*e.g.*, *CDK1C*)<br>2. Mosaicism<br>3. Chromosomal abnormalities<br>4. Molecular diagnosis not confirmed	DNA sequencing, MS PCR, karyotype

ICR1: imprinting control region 1; ICR2: imprinting control region 2; patUPD: paternal uniparental disomy; CNV: copy number variation; CMA: chromosome microarray analysis; SNP-CMA: single nucleotide polymorphism-CMA; MS PCR: methylation-specific polymerase chain reaction; STR: short tandem repeat; FISH: fluorescence *in situ* hybridization.
